# Arctigenin Attenuates Breast Cancer Progression through Decreasing GM-CSF/TSLP/STAT3/β-Catenin Signaling

**DOI:** 10.3390/ijms21176357

**Published:** 2020-09-02

**Authors:** Hui Shi, Luping Zhao, Xinlin Guo, Runping Fang, Hui Zhang, Guanjun Dong, Jia Fu, Fenglian Yan, Junfeng Zhang, Zhaochen Ning, Qun Ma, Zhihua Li, Chunxia Li, Jun Dai, Chuanping Si, Huabao Xiong

**Affiliations:** 1 Institute of Immunology and Molecular Medicine, Jining Medical University, Jining 272067, China; 8858shihui@mail.jnmc.edu.cn (H.S.); zhanghui1024@mail.jnmc.edu.cn (H.Z.); guanjun0323@mail.jnmc.edu.cn (G.D.); fujia730511@163.com (J.F.); yflian1117@mail.jnmc.edu.cn (F.Y.); zjfart001@163.com (J.Z.); ningzc@mail.jnmc.edu.cn (Z.N.); maqun@mail.jnmc.edu.cn (Q.M.); coco6016@mail.jnmc.edu.cn (Z.L.); xiachun1113@mail.jnmc.edu.cn (C.L.); immunedai@mail.jnmc.edu.cn (J.D.); 2 Institute of Basic Medical College, Jining Medical University, Jining 272067, China; zpersistence@163.com (L.Z.); gxlupupup@163.com (X.G.); 3 State Key Laboratory of Medicinal Chemical Biology, Department of Biochemistry, College of Life Sciences, Nankai University, Tianjin 300071, China; rpfang@163.com

**Keywords:** Arctigenin, GM-CSF, TSLP, STAT3, β-catenin, breast cancer

## Abstract

Invasive breast cancer is highly regulated by tumor-derived cytokines in tumor microenvironment. The development of drugs that specifically target cytokines are promising in breast cancer treatment. In this study, we reported that arctigenin, a bioactive compound from *Arctium lappa* L., could decrease tumor-promoting cytokines GM-CSF, MMP-3, MMP-9 and TSLP in breast cancer cells. Arctigenin not only inhibited the proliferation, but also the invasion and stemness of breast cancer cells via decreasing GM-CSF and TSLP. Mechanistically, arctigenin decreased the promoter activities of GM-CSF and TSLP via reducing the nuclear translocation of NF-κB p65 which is crucial for the transcription of GM-CSF and TSLP. Furthermore, arctigenin-induced depletion of GM-CSF and TSLP inhibited STAT3 phosphorylation and β-catenin signaling resulting in decreased proliferation, invasion and stemness of breast cancer cells in vitro and in vivo. Our findings provide new insights into the mechanism by which tumor-promoting cytokines regulate breast cancer progression and suggest that arctigenin is a promising candidate for cytokine-targeted breast cancer therapy.

## 1. Introduction

Breast cancer is considered the most common cancer for women worldwide and it is now the second leading cause of cancer-related deaths among females in the world [1,2]. Current treatments for breast cancer are focused mainly on surgical intervention, radiation therapy or a combination of these options based locally therapy with anti-cancer drugs, including chemotherapy, hormonal therapy and targeted therapy [3]. However, the comprehensive therapeutic strategies are still limited and a number of patients eventually develop into more aggressive malignant forms that are resistant to the most common treatments [4]. Accumulating evidence raised the potential role of the treatment-inducible cytokines in contributing to therapeutic resistance in breast cancer [5]. The network of cytokines activates multiple cell signaling pathways to promote tumor cell survival, proliferation, invasion, and migration [6,7]. Tumor-derived cytokines within the inflammatory microenvironment act to condition tissues for tumor colonization and proliferation via various mechanisms [8]. Numerous tumor-derived cytokines including granulocyte macrophage colony-stimulating factor (GM-CSF), thymic stromal lymphopoietin (TSLP), matrix metalloproteinase 3 (MMP-3) and matrix metalloproteinase 9 (MMP-9) have been reported to contribute to breast cancer progression [9,10,11]. Given that the functions of tumor-derived cytokines span multiple tissues and biological processes, these cytokines potently serves as new and important therapeutic targets in breast cancer [12]. Thus, searching for less toxic but effective therapeutics targeting these cytokines is urgently needed.

STAT3 pathway and β-catenin signaling are frequently dysregulated in breast cancer, contributing to cancer stem cells (CSCs)-associated tumor initiation, angiogenesis, metastasis, therapy resistance, and tumor relapse [13,14]. In breast cancer, CSCs are often regulated by complex interactions with the components of the tumor microenvironment through networks of tumor-derived cytokines [15].

Arctigenin is an active ingredient isolated from the seeds of *Arctium lappa* L. (Asteraceae) which is a medicinal herb widely distributed in China, Japan and Korea [16]. Arctigenin has been reported to possess several pharmacological activities, including anti-oxidant, anti-inflammatory and anti-tumor [17,18,19]. In several types of tumor, arctigenin has been reported to exert anti-tumor effects via inhibiting the nuclear translocation of NF-κB [20]. In breast cancer, arctigenin induces apoptosis of breast cancer cells and confers anti-metastatic effects [21,22,23]. Since it is well accepted that arctigenin is efficient in breast cancer, whether arctigenin modulates tumor-derived cytokines and the underlying mechanism have not been investigated.

In the present study, the effect of arctigenin on tumor-derived cytokines in the microenvironment of breast cancer was investigated. The results uncovered that arctigenin attenuated the progression of breast cancer through decreasing tumor-derived GM-CSF and TSLP. Mechanistically, arctigenin decreased the promoter activities of GM-CSF and TSLP via hindering the nuclear translocation of NF-κB p65, and consequently, inhibited STAT3/β-catenin signaling, resulting in decreased proliferation, invasion and stemness of breast cancer cells in vitro and in vivo.

## 2. Results

### 2.1. Arctigenin Attenuates Breast Cancer Growth In Vivo

**Figure 1:**
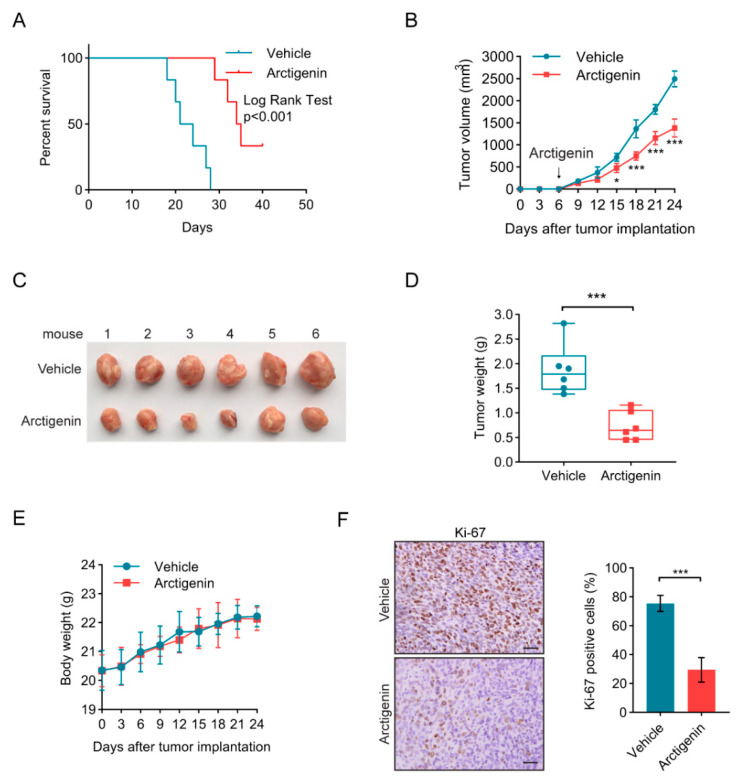
Arctigenin attenuates breast cancer growth in vivo. (**A**) Kaplan–Meier plot of survival of 4T1 tumor-bearing mice treated with vehicle or arctigenin. n = 6 mice/group, *p* < 0.001 (Log Rank test). (**B**) The growth curve of 4T1 tumors in mice treated with arctigenin or vehicle. Arrows indicated the start day of arctigenin administration. (**C**) Photograph of the tumors separated from mice in each group. (**D**) The tumor weight of 4T1 tumors treated with arctigenin or vehicle. (**E**) The body weight of mice treated with arctigenin or vehicle. (**F**) Representative Ki-67 staining (left panel) and statistics (right panel) of the 4T1 tumor sections. Scale bar, 100 μm. For (**B**,**D**,**E**), data represent mean ± SD from six mice. Statistically significant differences are indicated: * *p* < 0.05, *** *p* < 0.001 (Student’s *t* test).

To investigate the anti-tumor effect of arctigenin on breast cancer in vivo, a 4T1 orthotopic breast cancer mouse model was used. Arctigenin intervention prolonged the survival of 4T1 tumor-bearing mice (Figure 1A). Meanwhile, arctigenin significantly hindered tumor growth (Figure 1B) and reduced tumor size and weight (Figure 1C,D) with no major effect on body weight, suggesting that arctigenin treatment would not lead to significant cytotoxicity in vivo (Figure 1E). Immunohistochemistry demonstrated that arctigenin treatment markedly reduced the expression of Ki-67 in tumor tissues which is associated with cellular proliferation (Figure 1F). Together, these data suggest that arctigenin effectively attenuates breast cancer progression in vivo.

### 2.2. Arctigenin Suppresses GM-CSF and TSLP Expression in Breast Cancer Cells

**Figure 2:**
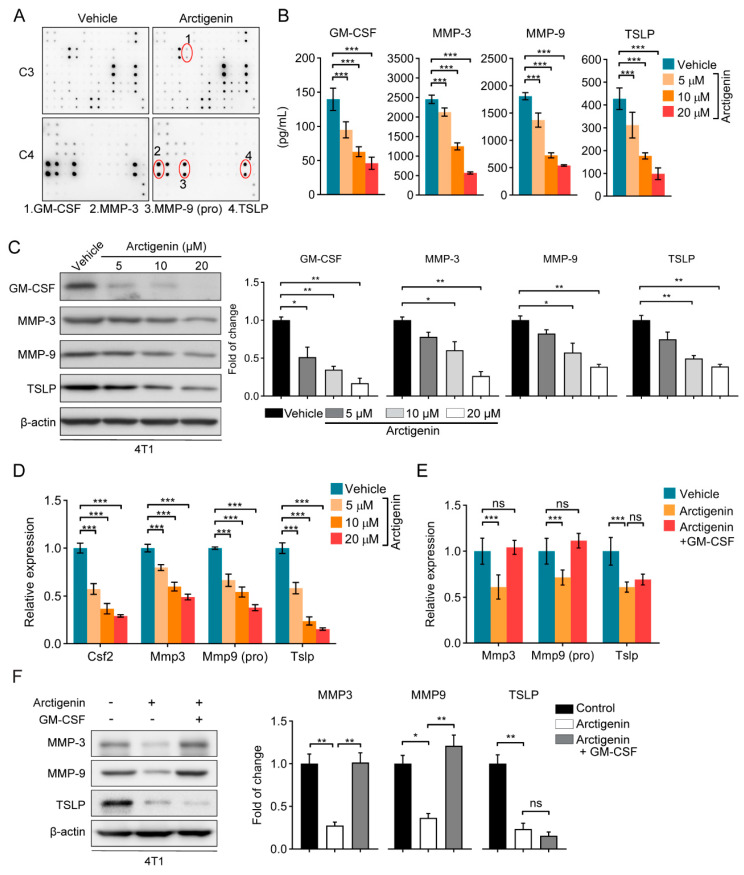
Arctigenin suppresses GM-CSF and TSLP expression in breast cancer cells. (**A**) Mouse cytokine array analysis of conditioned medium from 4T1 cells treated with arctigenin (10 μM) or vehicle. (**B**) Quantification of GM-CSF, MMP-3, MMP-9 and TSLP in conditioned medium from 4T1 cells in the presence or absence of indicated concentrations of arctigenin. (**C**) Western blot analysis of GM-CSF, MMP-3, MMP-9 and TSLP in 4T1 cells treated with arctigenin at indicated concentration. (**D**) qRT-PCR analysis of the expressions of GM-CSF, MMP-3, MMP-9 (pro) and TSLP in 4T1 cells treated with indicated concentrations of arctigenin. (**E**,**F**) qRT-PCR analysis of the expressions of MMP-3, MMP-9 (pro) and TSLP (**E**) and Western blot analysis of the expressions of MMP-3, MMP-9 and TSLP (**F**) in 4T1 cells treated with recombinant mouse GM-CSF (1 ng/mL) in the presence of arctigenin (20 μM). For (**B**–**F**), values represent mean ± SEM of three independent experiments. Statistically significant differences are indicated: * *p* < 0.05, ** *p* < 0.01, *** *p* < 0.001, ns, not significant, *p* > 0.05 (One-way ANOVA).

Tumor-derived cytokines modulate the tumor microenvironment and play important roles for tumor progression [24,25]. To examine whether arctigenin regulates tumor-related cytokines, we analyzed the conditioned medium of 4T1 cells treated with arctigenin using cytokine array. Arctigenin intervention could decrease GM-CSF, MMP-3, MMP-9 and TSLP levels in conditioned medium (Figure 2A and Supplementary Table S2). Additionally, these results were further confirmed by ELISA using the conditioned medium of 4T1 cells or MDA-MB-231 cells treated with vehicle or arctigenin. Arctigenin could dose dependently decrease the levels of GM-CSF, MMP-3, MMP-9 and TSLP in the conditioned medium (Figure 2B and Supplementary Figure S1A). Furthermore, arctigenin intervention suppressed protein expressions and mRNA level of GM-CSF, MMP-3, MMP-9 and TSLP in breast cancer cells (Figure 2C,D and Supplementary Figure S1B), suggesting that arctigenin decreases the secreted GM-CSF, MMP-3, MMP-9 and TSLP at the transcriptional level in breast cancer cells. Previous studies reported that MMP-3 is an activator for the activity of MMP-9 which is a transcriptional target of GM-CSF [26,27]. Thus, we investigated whether arctigenin regulated MMP-3 and MMP-9 expression through GM-CSF. Recombinant mouse GM-CSF could completely rescue the expression of MMP-3 and MMP-9 at both mRNA and protein levels, but not TSLP (Figure 2E,F), suggesting that GM-CSF contributed to arctigenin-regulated MMP-3 and MMP-9 expression. Taken together, arctigenin could inhibit the expressions of GM-CSF and TSLP in breast cancer cells.

### 2.3. Arctigenin Decreases the Promoter Activities of GM-CSF and TSLP through Inhibiting the Nuclear Translocation of NF-κB p65

**Figure 3:**
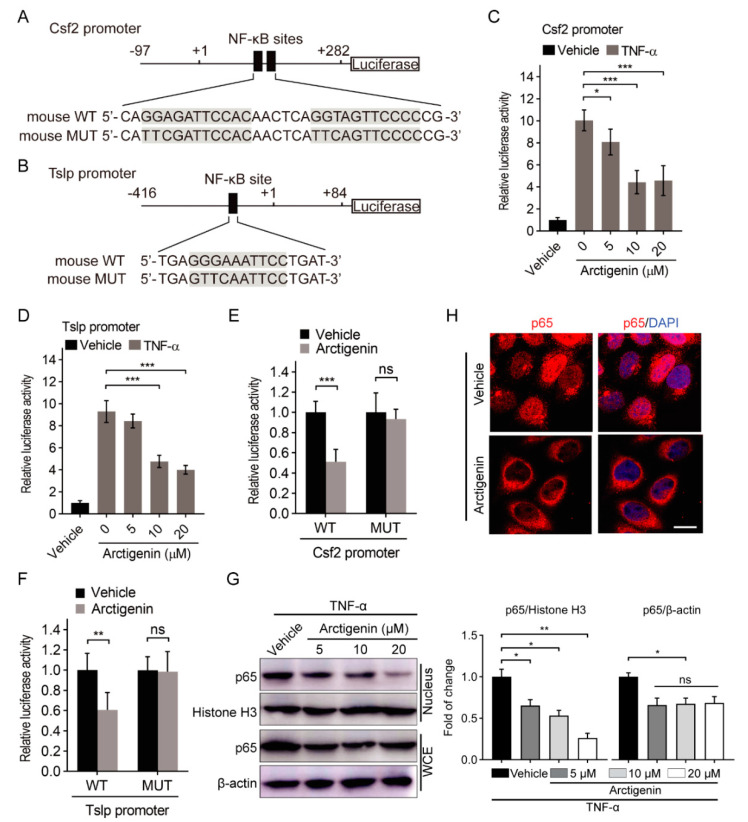
Arctigenin decreases the promoter activities of GM-CSF and TSLP through inhibiting the nuclear translocation of NF-κB p65. (**A**,**B**) Wild type (**A**) or mutated (**B**) NF-κB binding site in the constructed mouse Csf2 and Tslp promoters. Mutant sequence was highlighted by gray box. (**C**,**D**) The promoter activities of mouse Csf2 (**C**) and Tslp (**D**) in TNF-α-stimulated 4T1 cells treated with vehicle or arctigenin were measured by luciferase reporter gene assays. (**E**,**F**) The activities of wild-type (WT) or mutated (MUT) mouse Csf2 promoters (**E**) and Tslp promoters (**F**) were measured by luciferase reporter gene assays. (**G**) Western blot analysis of p65 expression in the nucleus and whole cell extract (WCE) of TNF-α-stimulated 4T1 cells. (**H**) Representative immunofluorescence staining of p65 in TNF-α-stimulated 4T1 cells treated with vehicle or arctigenin. DAPI counterstaining (blue) are used to indicate nucleus. Scale bar, 20 μm. For (**C**,**D**,**G**), * *p* < 0.05, *** *p* < 0.001 (One-way ANOVA). For (**E**,**F**), ns, not significant, *p* > 0.05, ** *p* < 0.01, *** *p* < 0.001 (Student’s *t* test). Values represent mean ± SEM of three independent experiments.

It has been reported that there are NF-κB binding sites in the promoter of GM-CSF and TSLP [28,29], and arctigenin could inhibit NF-κB activation through suppressing nuclear translocation of p65 [30]. Thus, arctigenin might inhibit the promoters of GM-CSF and TSLP by suppressing NF-κB activation. To explore the mechanism underlying arctigenin affected expressions of GM-CSF and TSLP, we constructed the minimal promoters of GM-CSF and TSLP separately according to previous studies (Figure 3A,B) [30,31]. Dual-Luciferase reporter assay showed that arctigenin reduced TNF-α-stimulated promoter activities of GM-CSF and TSLP in 4T1 cells (Figure 3C,D). Furthermore, we mutated the NF-κB binding sites in the promoters of GM-CSF and TSLP (Figure 3A,B). NF-κB binding site mutation abolished the effect of arctigenin on the promoter activities of GM-CSF and TSLP in 4T1 cells (Figure 3E,F). Considering that TNF-α can activate NF-κB through promoting the nuclear translocation of p65 [32], we explored the effect of arctigenin on p65 nuclear translocation. Arctigenin was able to decrease TNF-α-stimulated nuclear expression and localization of p65 in 4T1 cells (Figure 3G,H), suggesting that arctigenin inhibits p65 nuclear translocation. These results indicate that arctigenin decreases the promoter activities of GM-CSF and TSLP through inhibiting NF-κB activation.

### 2.4. Arctigenin Inhibits Breast Cancer Cell Proliferation, Invasion and Stemness through Decreasing GM-CSF and TSLP

**Figure 4:**
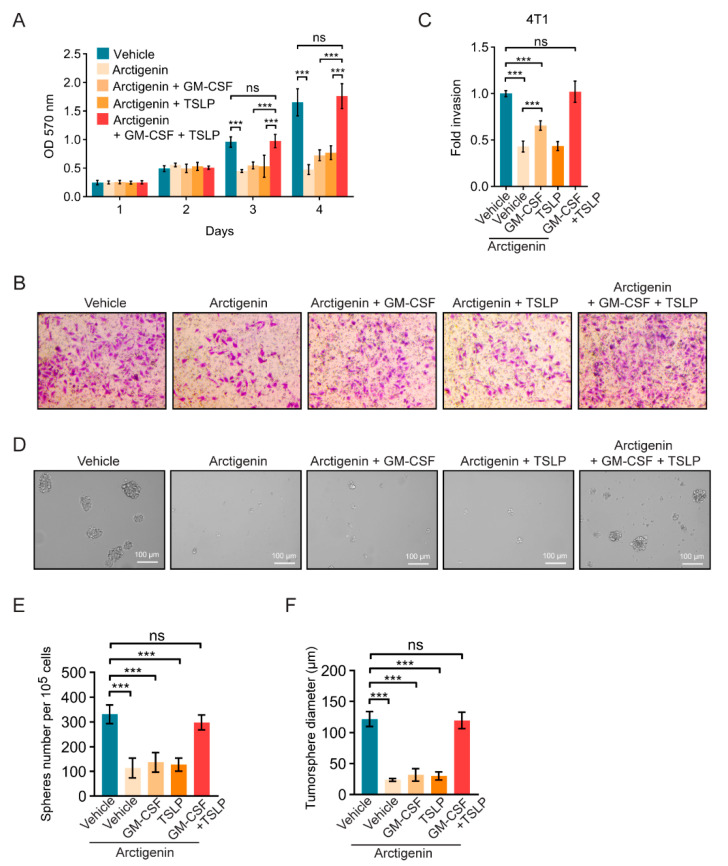
Arctigenin inhibits breast cancer cells proliferation, invasion and stemness through decreasing GM-CSF and TSLP. (**A**) Cell proliferation of 4T1 cells treated with arctigenin (20 μM) in the presence or absence of GM-CSF (1 ng/mL) and/or TSLP (100 ng/mL). (**B**,**C**) Representative images (**B**) and statistics (**C**) of the invaded 4T1 cells treated with arctigenin (20 μM) in the presence or absence of GM-CSF and TSLP. (**D**) Representative images of the tumorsphere formation of 4T1 cells in the presence or absence of GM-CSF and TSLP. (**E**,**F**) Quantification of tumorspheres formation efficiency (**E**) and tumorsphere diameter (**F**). For (**A**,**C**,**E**,**F**), ns, not significant, *p* > 0.05, *** *p* < 0.001 (One-way ANOVA). Values represent mean ± SEM of three independent experiments.

Next, the roles of GM-CSF and TSLP in arctigenin-regulated anti-tumor effect in breast cancer cells was investigated. Arctigenin significantly suppressed the proliferation and invasion of 4T1 or MDA-MB-231 cells, however, the supplement of recombinant mouse GM-CSF or TSLP could partially reverse arctigenin-mediated anti-proliferation or anti-invasion effect (Figure 4A–C and Supplementary Figure S2A–C). Notably, the supplement of GM-CSF and TSLP completely rescue the proliferation and invasion of 4T1 or MDA-MB-231 cells (Figure 4A–C and Supplementary Figure S2A–C), suggesting that arctigenin inhibits breast cancer cell proliferation and invasion via GM-CSF and TSLP. It has been suggested that breast cancer stem cells are resistant to many conventional therapeutic approaches, because traditional approaches might kill the majority of tumor cells, some of the breast cancer stem cells remain unaffected, surviving and generating new tumors [33,34]. To examine whether arctigenin could affect the stemness of breast cancer cells, we conducted tumorsphere formation assays using breast cancer cells treated with arctigenin. The tumorsphere forming assay is considered to demonstrate the self-renewing ability of stem cells. Arctigenin significantly reduced tumorsphere forming efficiency (TFE) and tumorsphere diameter of 4T1 and MDA-MB-231 cells (Figure 4D–F and Supplementary Figure S2D–F). The effects of arctigenin on tumorspheres could be completely rescued by GM-CSF and TSLP (Figure 4D–F and Supplementary Figure S2D–F). These results imply that arctigenin not only inhibits the proliferation and invasion but also the stemness of breast cancer cells through decreasing GM-CSF and TSLP.

### 2.5. Arctigenin Inhibits STAT3/β-Catenin Signaling through Decreasing GM-CSF and TSLP

**Figure 5:**
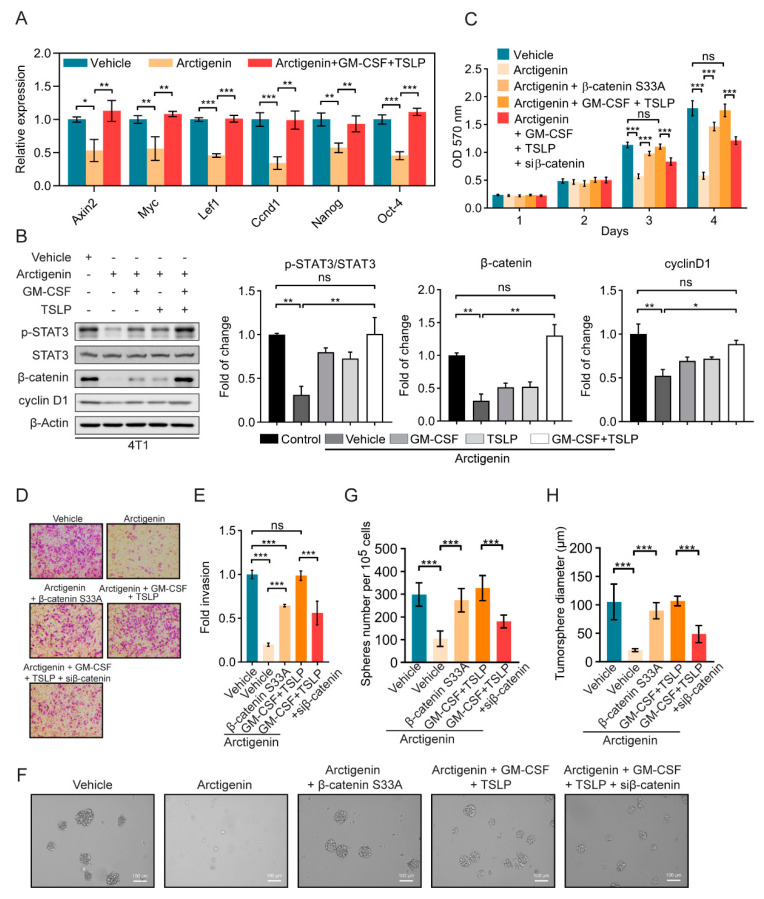
Arctigenin inhibits STAT3/β-catenin signaling through decreasing GM-CSF and TSLP. (**A**) The expression of β-catenin downstream genes Axin2, Myc, Lef1, Ccnd1, Nanog and Oct4 were analyzed by qRT-PCR with the indicated treatments. (**B**) The expression levels of phosphorylated STAT3 (p-STAT3), STAT3, β-catenin and cyclin D1 were analyzed by Western blotting with the indicated treatments. (**C**) Cell proliferation analysis of 4T1 cells treated with arctigenin (20 μM) in the presence or absence of GM-CSF (1 ng/mL), TSLP (100 ng/mL), or transfectd with β-catenin S33A construct or siRNA of β-catenin. (**D**,**E**) Representative images (**D**) and (**E**) statistics of the invaded 4T1 cells with the indicated treatments. (**F**) Representative images of the tumorsphere in 4T1 cells after indicated treatments. (**G**,**H**) Quantification of tumorspheres formation efficiency (**G**) and tumorsphere diameter (**H**). For (**A**–**C**,**E**,**G**,**H**), ns, not significant, *p* > 0.05, * *p* < 0.05, ** *p* < 0.01, *** *p* < 0.001 (One-way ANOVA). Values represent mean ± SEM of three independent experiments.

It has been reported that GM-CSF and TSLP are important players in STAT3 activation [35,36]. And the crosstalk between β-catenin and STAT3 signaling pathways has been reported in breast cancer stem cells [37]. Additionally, Zheng Lu et al. reported that arctigenin inhibits hepatocellular carcinoma metastasis via suppressing β-catenin signaling [38]. These studies raise question that whether arctigenin could regulate β-catenin signaling and the crosstalk between β-catenin signaling and STAT3 signaling, which regulates the stemness of breast cancer cells. To answer the question, the role of GM-CSF and TSLP in the regulation of β-catenin downstream genes was first investigated using qRT-PCR. Strikingly, arctigenin could inhibit the expression of β-catenin downstream genes, and supplement of GM-CSF and TSLP rescued their expression (Figure 5A). Furthermore, the expression of β-catenin and its downstream gene cyclinD1, and the activation of STAT3 was determined by Western blotting. GM-CSF or TSLP treatment could partially rescue the expression of β-catenin and cyclinD1 (Figure 5B and Supplementary Figure S3A), while dual-treatment with GM-CSF and TSLP completely rescued the expression of β-catenin and cyclinD1 (Figure 5B and Supplementary Figure S3A), suggesting that GM-CSF and TSLP are playing important roles in the arctigenin-inhibited expression of β-catenin. The expression of β-catenin S33A, a frequently occurring site mutation of β-catenin in cancers [39], could partially rescue the proliferation and invasion of breast cancer cells treated with arctigenin (Figure 5C–E and Supplementary Figure S3B,C), while GM-CSF and TSLP dual treatment could completely rescue the proliferation and invasion (Figure 5C–E and Supplementary Figure S3B,C). Additionally, knockdown of β-catenin attenuated proliferation and invasion when GM-CSF and TSLP were present in the culture medium of 4T1 and MDA-MB-231 cells (Figure 5C–E and Supplementary Figure S3B,C), suggesting that GM-CSF and TSLP rescued arctigenin-inhibited proliferation and invasion through β-catenin. Interestingly, β-catenin S33A could completely rescue tumorsphere formation of 4T1 and MDA-MB-231 cells, while β-catenin knockdown decreased both tumorsphere formation efficiency and tumorsphere diameter (Figure 5F–H and Supplementary Figure S3D–F), suggesting that β-catenin contributed to stemness of breast cancer cells which is regulated by arctigenin. Taken together, arctigenin inhibits STAT3/β-catenin signaling through decreasing GM-CSF and TSLP.

### 2.6. Arctigenin Inhibits Breast Cancer Progression via Decreasing GM-CSF and TSLP In Vivo

**Figure 6:**
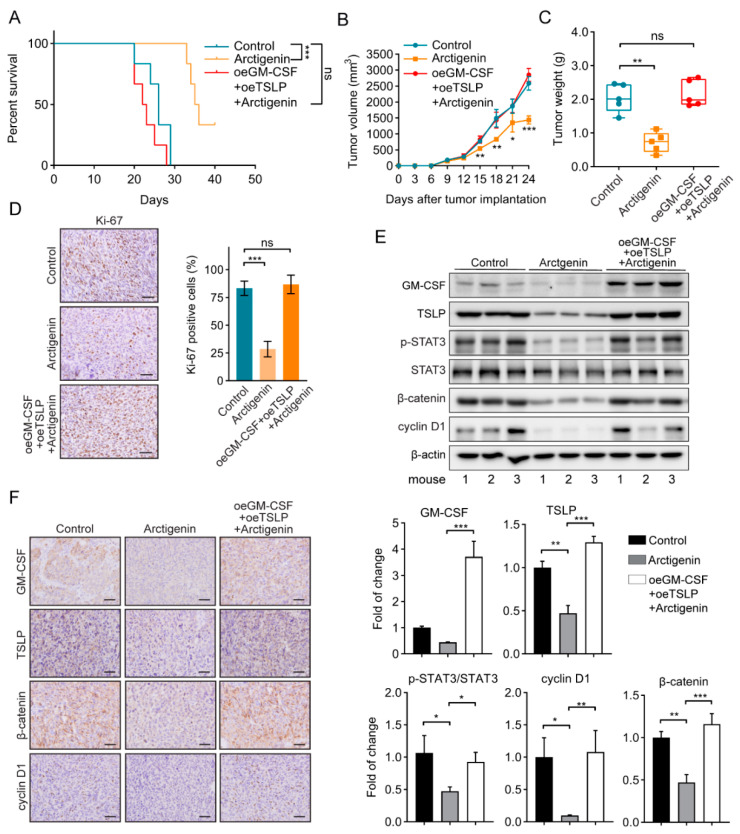
Arctigenin inhibits breast cancer progression via decreasing GM-CSF and TSLP in vivo. (**A**) Kaplan–Meier survival curves for mice injected with the indicated 4T1 cells and/or treated with arctigenin. oe, overexpression. n = 6 mice/group. *** *p* < 0.01, ns, not significant, *p* > 0.05 (Log Rank test). (**B**) The tumor volume of 4T1 tumors separated from mice of each group. (**C**) The tumor weight of separated 4T1 tumors in each group. (**D**) Representative Ki-67 staining (left panel) and statistics (right panel) of 4T1 tumors from the indicated mice. Scale bar, 100 μm. (**E**) The levels of GM-CSF, TSLP, p-STAT3, STAT3, β-catenin and cyclin D1 of the indicated tumors from representative mice were analyzed by Western blotting analysis. Mouse number was indicated at bottom. (**F**) Representative immunohistological staining of GM-CSF, TSLP, β-catenin and cyclin D1 in the indicated tumor sections. Scale bar, 100 μm. (**G**) Schematic diagram of working mechanism by which arctigenin suppresses breast cancer progression. For (**B**–**D**) (right panel) and (**E**), the data are shown as mean ± SD. Statistically significant differences are indicated: * *p* < 0.05, ** *p* < 0.01, *** *p* < 0.001, ns, not significant, *p* > 0.05 (One-way ANOVA).

Finally, we sought to investigate the effects of GM-CSF and TSLP on arctigenin-inhibited breast cancer progression in vivo. Overexpression of GM-CSF and TSLP made arctigenin failed to elongate the survival of tumor-bearing mice (Figure 6A), failed to reduce tumor volume and weight (Figure 6B,C). Ki-67 staining showed that overexpression of GM-CSF and TSLP made tumor sustained proliferation even if arctigenin was administrated (Figure 6D). Consequently, the expression of GM-CSF, TSLP, phosphorylated STAT3, β-catenin, and cyclin D1 in the transplanted tumors was evaluated using Western blotting (Figure 6E), and the expression of GM-CSF, TSLP, β-catenin and cyclin D1 was examined in tumor sections (Figure 6F). Collectively, we conclude that arctigenin inhibits the progression of breast cancer by reducing GM-CSF and TSLP expression.

## 3. Discussion

**Figure 7:**
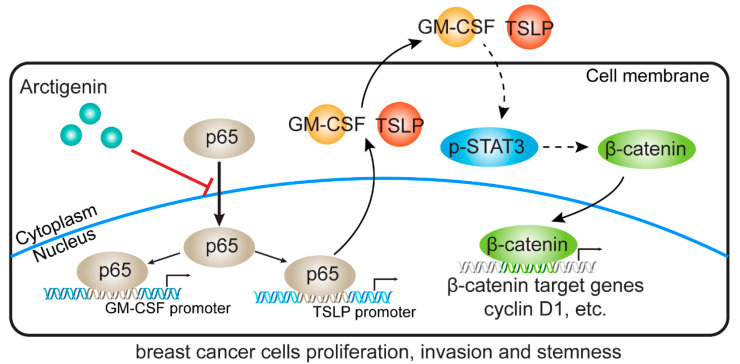
Schematic diagram of working mechanism by which arctigenin suppresses GM-CSF and TSLP in breast cancer. Arctigenin decreased the promoter activities of GM-CSF and TSLP via hindering the nuclear translocation of NF-κB p65 which is crucial for the transcription of GM-CSF and TSLP. Arctigenin-induced depletion of GM-CSF and TSLP inhibited STAT3 phosphorylation and β-catenin signaling resulting in decreased proliferation, invasion and stemness of breast cancer cells. T-arrows, negative regulation; solid arrows, direct positive regulation; dashed arrows, indirect positive regulation.

For many years clinicians and researchers are examining and exploring various therapeutic modalities for breast cancer [40]. Yet the disease has not been conquered and the development of new targeted therapy is still going on. Natural products are major sources providing due to their potentially low toxicity and potential effectiveness, but the functional mechanism of most of them remain unclear [41,42]. The present study showed that arctigenin reduced the expressions of tumor-derived GM-CSF and TSLP through NF-κB p65 to inhibit breast cancer progression. Decreased GM-CSF and TSLP resulted in the mute of STAT3/β-catenin signaling and the suppression of breast cancer progression (Figure 7).

In recent years, accumulating evidences have been raised to support the role of cytokines in breast cancer. New signaling pathways of interferon regulatory factors 1 (IRF-1) and 2 (IRF-2) and interleukin (IL)-1 family, IL-6, IL-11, IL-18, and interferons (IFNs) have been found within tumor microenvironments. Some cytokines (IL-1, IL-6, IL-11) stimulate while others (IL-12, IL-18, IFNs) inhibit breast cancer proliferation and/or invasion [43]. IL7, IL11 and IL17 promotes growth and proliferation and associates with worse prognosis [44]. IL-20 enhances cell proliferation through cathepsin K and cathepsin G. TNF-α promotes inflammatory microenvironment and upregulates proliferation. TNF-α also increases the expression of oncogenes, such as MMPs, IL-8, CXCR, VEGF, and MCP-1 and induces the activation of several pathways such as NF-κB, PI3K/AKT, Ras/Raf/MEK1/ERK, GSK-3b [45]. In the early stages of tumorigenesis, TGFβ functions as a tumor suppressor, but in later stages, tumor cells will escape from this effect and progress in response to TGF-b [46]. So far IL-2, IFNα, IFNβ and occasionally IFNγ, IL-6, IL-12 have been the cytokines used for anti-tumor treatment of advanced breast cancer either to induce or increase hormone sensitivity and/or to stimulate cellular immunity. Disappointing results occurred in most trials [43]. Through analyzing the effect of arctigenin on tumor-derived cytokines, we found that the cytokines GM-CSF, MMP-3, MMP-9 and TSLP contribute not only cell proliferation and invasion, but also cell stemness of breast cancer, which extends the landscape of cytokines function in breast cancer progression.

The anti-carcinogenic activity of arctigenin has been demonstrated in vitro and in a few animal studies in several cancers, including breast, pancreatic and lung cancer, associated with the induction of apoptosis, inhibition of proliferation and modulations of multiple signaling pathways [47,48,49,50]. In the present study, the anti-tumor effect of arctigenin was first evaluated by an orthotopic breast cancer mouse model which revealed that arctigenin could reduce tumor growth and elongate the survival of tumor-bearing mice.

Tumor progression is accompanied by the production of a wide range of factors by both tumor and non-tumor cells which form tumor microenvironment and benefit tumor growth in return [24]. Tumor-derived cytokines play important roles in breast cancer progression and could be therapeutic targets [51]. Our finding indicated that arctigenin decreased the expression of tumor-derived cytokines, including GM-CSF, MMP-3, MMP-9 and TSLP. GM-CSF is an important cytokine regulating STAT3 activation [52]. High levels of MMPs family are involved in invasion, angiogenesis, and osteolysis in breast cancer [53]. It has been reported that 4T1 cells produce TSLP and the level of TSLP expression is correlated with tumor growth and metastasis [10]. The phosphorylation of STAT3 occurred upon TSLP stimulation [54]. The present study supported that arctigenin suppressed breast cancer progression through the inhibition of GM-CSF, MMP-3, MMP-9, and TSLP. Previous studies suggest that NF-κB accounts for the transcription of GM-CSF and TSLP [28,29]. Besides, arctigenin is reported to inhibit the nuclear translocation of NF-κB [55]. Consistently, arctigenin was identified to suppress GM-CSF and TSLP transcription through inhibiting the nuclear translocation of NF-κB p65 in the current study.

As breast cancer stem cells (BCSCs) are dormant in nature, it is highly likely that they fail to directly respond to the drugs which are meant for ablating rapidly proliferating cells [56]. As a result, tumor resistance and recurrence eventually occur. It would be important to target BCSCs for cancer treatment [57]. The present study supported that arctigenin inhibited not only proliferation and invasion but also tumorsphere formation of breast cancer cells which is reflecting stemness. β-catenin signaling is found to influence the cell fate of BCSCs [58]. While, STAT3 pathway is another crucial player involves in the maintenance, growth and viability of cancer stem cells. Recent study indicated that the crosstalk between STAT3 pathway and β-catenin signaling contributes to cancer stem cells [59]. While, arctigenin decreased the expression of GM-CSF and TSLP and muted STAT3/β-catenin signaling in breast cancer cells, resulting in inhibited self-renewal ability of BCSCs.

In summary, we uncovered that arctigenin exerted anti-tumor effect through decreasing tumor-derived GM-CSF and TSLP, and targeting the proliferation, invasion and stemness of breast cancer cells. Arctigenin inhibited the promoter activities of GM-CSF and TSLP via hindering the nuclear translocation of transcription factor p65. Consequently, the decrease of GM-CSF/TSLP muted STAT3/β-catenin signaling and inhibited tumor progression. These findings provide new insights into the mechanism by which tumor-derived cytokines regulated cancer progression, consolidate the crosstalk between STAT3 and β-catenin signaling, and raise the possibility that arctigenin acts as an inhibitor of tumor-derived GM-CSF and TSLP for the treatment of breast cancer.

## 4. Materials and Methods

### 4.1. Mouse Model

BALB/c female mice were purchased from Animal Model Research Institute of Nanjing University (Nanjing, China) and housed in a pathogen-free barrier facility in a 12 h light/dark cycle. All procedures were approved by the Committee on the Ethics of Animal Experiments of Jining Medical University (protocol approval number 2018-JC-012). To establish tumor model, 50 µL of cell suspension (5 × 10^5^ 4T1 cells) were injected into the 3rd mammary pad of female mice. Then, all mice were randomly divided into groups. Mice were intragastrically administrated with 50 mg/kg arctigenin (Sigma-Aldrich, Saint Louis, MO, USA) daily, whereas mice received an equal volume of vehicle (2% DMSO in corn oil) as control. Tumor volume was monitored every three days and was calculated by the formula: volume = 1/2 × length × (width)^2^. At the end of the study period (24 days after tumor cell injection), six mice from each group were randomly selected for weighing and excising. The remaining six mice for each group were kept in standard conditions and fed with standard diet, had free access to water, and maintained in a 12 h light/dark cycle until they died. Daily deaths were recorded, and after the last death in both groups, data were analyzed with a Kaplan–Meier test.

### 4.2. Cell Culture and Transfection

Murine mammary cancer cell line 4T1 and the human breast cancer cell line MDA-MB-231 were obtained from the Chinese Academy of Science Cell Bank (Shanghai, China). The cells were maintained in Dulbecco’s Modified Eagle’s medium (DMEM, Gibco, CA, USA) supplemented with heat-inactivated 10% fetal bovine serum (FBS, Gibco, CA, USA), 100 U/mL penicillin (Sigma-Aldrich, Saint Louis, MO, USA) and 100 μg/mL streptomycin (Sigma-Aldrich, Saint Louis, MO, USA) and grown at 37 °C with 5% CO_2_. Cells were treated with 5 μM, 10 μM or 20 μM arctigenin (Sigma-Aldrich, Saint Louis, MO, USA), 1 ng/mL GM-CSF (BioLegend, San Diego, CA, USA), 100 ng/mL TSLP (R&D systems, Minneapolis, MN, USA) or equal volume of vehicle before further analysis. All transfections were performed using Lipofectamine 2000 reagent (Invitrogen, Thermo Fisher Scientific, Waltham, MA, USA) according to the manufacturer’s instruction. β-catenin siRNAs were synthesized by Sigma, and the sequences were as follows: (mouse) siCTNNB1-sense: AAGUCCUGUAUGAGUGGGAAC; siCTNNB1-antisense: GUUCCCACUCAUACAGGACUU; (human) siCTNNB1-sense: AAGUCCUGUAUGAGUGGGAAC; siCTNNB1-antisense: GUUCCCACUCAUACAGGACUU.

### 4.3. Immunostaining

Immunohistochemical staining was performed according to standard protocol. For immunofluorescent staining, cells were fixed in 4% paraformaldehyde, permeabilized with 0.05% Triton X-100 (Sigma-Aldrich, Saint Louis, MO, USA), and blocked with 3% BSA (w/v) (Sigma-Aldrich, Saint Louis, MO, USA). Nuclei were counterstained with DAPI (Sigma-Aldrich, Saint Louis, MO, USA). Fluorescent micrographs were obtained using a confocal microscopy (Leica TCSSP5, Buffalo Grove, IL, USA). The antibodies used were summarized in Supplementary Table S1.

### 4.4. Cytokine Antibody Array

Mouse Cytokine Array C1000 (RayBiotech, Peachtree Corners, GA, USA) was used for the screening of arctigenin targets. 4T1 cells were plated at equal number and cultured for 48 h. Conditioned medium were collected and processed according to the manufacturer’s instruction. A luminescence detector (Imager600, General Electric Company, New York, NY, USA) was used for the detection of signal intensity. The characterization of the Mouse Cytokine Array C1000 was summarized in Supplementary Table S2.

### 4.5. Quantitative Reverse-Transcription Polymerase Chain Reaction (qRT-PCR)

qRT-PCR was performed by a Bio-Rad sequence detection system according to the manufacturer’s instructions using double-stranded DNA-specific SYBR Green Premix Ex Taq™ II Kit (TaKaRa Bio, Beijing, China). Experiments were conducted in duplicate in three independent experiments. The primers used for qRT-PCR were listed in Supplementary Table S3.

### 4.6. Western Blot Analysis

Western blotting was performed according to standard protocol. The antibodies used were summarized in Supplementary Table S1.

### 4.7. Enzyme-Linked Immunosorbent Assay (ELISA)

The cytokines in 4T1 cells or MDA-MB-231 cells conditioned medium were quantified using a competitive enzyme linked immunoassay according to the manufacturers’ instructions. The following cytokines were tested: GM-CSF (Cat#: DY415, DY215, R&D systems, Minneapolis, MN, USA), MMP-3 (Cat#: DY548, DMP300, R&D Systems, Minneapolis, MN, USA), MMP-9 (Cat#: DY6718, DY911, R&D Systems, Minneapolis, MN, USA), and TSLP (Cat#: DY555, DY1398, R&D Systems, Minneapolis, MN, USA).

### 4.8. Construction of Plasmids

The 5′-flanking region (from −97 to +282 nt) of mouse Csf2 (NC_000077 REGION: (54249617.. 54249996) and the 5′-flanking region (from −416 to +84 nt) of mouse Tslp (NC_000084 REGION: (32814967..32815466) was amplified and inserted into the upstream of fire fly luciferase gene in pGL3-Basic vector (Promega, Madison, WI, USA), named pGL3-Csf2 and pGL3-Tslp, respectively. The mutant constructs of pGL3-Csf2-MUT and pGL3-Tslp-MUT were carried out using QuikChange II XL Site-Directed Mutagenesis Kit (Agilent, Santa Clara, CA, USA). All primers were listed in Supplementary Table S3.

### 4.9. Luciferase Reporter Gene Assays

The promoter activities of Csf2 and Tslp were determined in 4T1 cells transfected with wild type or mutant of pGL3-Csf2 or pGL3-Tslp respectively following 6 h stimulation with 50 ng/mL TNF-α (BioLegend, San Diego, CA, USA) and 30 min pre-incubation with 20 μM arctigenin or vehicle. Luciferase reporter gene assays were implemented using Dual-Luciferase Reporter Assay System (Promega, Madison, WI, USA) according to the manufacturer’s instructions.

### 4.10. Nuclear Extract Isolation

Cells were lysed in the harvest buffer (10 mM HEPES, 50 mM NaCl, 0.5 M Sucrose, 0.1 mM EDTA, 0.5% Triton X-100, 1 mM DTT, pH 7.9). The lysates were centrifuged at 1000 rpm. Then, the pellets (nuclear extracts) were washed three times and then boiled in 1% SDS for Western blot analysis.

### 4.11. Cell Proliferation Assay

For the cell proliferation assay, 1 × 10^4^ cells were plated in 96-well plates. Cell growth was determined using a standard tetrazolium bromide (MTT) assay (Sigma-Aldrich, Saint Louis, MO, USA) at the indicated time points. Data represent the means ± SEM of three independent experiments.

### 4.12. Boyden Chamber Invasion Assay

Invasion assays were carried out in 24-well plate cell culture inserts (8 μm) (Corning, New York, NY, USA) pre-coated with Matrigel Matrix with growth factor reduced at the concentration of 1-2 mg/mL (BD Biosciences, Franklin Lakes, NJ, USA). Cells were seeded at 2 × 10^5^ cells per well in serum-free medium. The cells were induced to invade toward medium containing 10% FBS for 24 h in cell culture incubator. The invaded cells were stained with crystal violet. For quantitative analysis, the crystal violet in the cells was released using 10% acetic acid and the optical density of the 10% acetic acid was read at 595 nm using a microplate reader. For quantitative analysis compare the data with data from a serial dilution series.

### 4.13. Tumorsphere Formation

Tumorsphere culture was performed in low attachment dishes (Corning, New York, NY, USA), supplemented with B27 (Invitrogen), 20 ng/mL epidermal growth factor (EGF), 20 ng/mL basic fibroblast growth factor (bFGF; Peprotech, Rocky Hill, NJ, USA) and 4 μg/mL heparin (Sigma-Aldrich, Saint Louis, MO, USA). Dishes were cultivated for seven days to enumeration of spheres. Individual spheres ≥100 μm from each replicate well (n ≥ 6 wells) were counted under an inverted microscope at 50× magnification using the Image-Pro Plus program (Media Cybernetics, Rockville, MD, USA).

### 4.14. Statistical Analysis

GraphPad Prism 7.0 was used for statistical analysis, Student’s *t*-test were used for unpaired data. One-way ANOVA was used for multiple comparisons. Statistical significance was assessed by comparing mean values (±SEM), *p* values less than 0.05 were considered significant. Each experiment was repeated at least three times.

## Supplementary Materials

Supplementary materials can be found at https://www.mdpi.com/1422-0067/21/17/6357/s1.

## Abbreviations

GM-CSF	Granulocyte macrophage colony-stimulating factor
TSLP	Thymic stromal lymphopoietin
MMP3	Matrix metalloproteinase 3
MMP9	Matrix metalloproteinase 9
CSC	Cancer stem cells
NF-κB	Nuclear factor kappa B
STAT3	Signal transducer and activator of transcription 3
BCSCs	Breast cancer stem cells
