# Correction: Shi, H., et al. Arctigenin Attenuates Breast Cancer Progression through Decreasing GM-CSF/TSLP/STAT3/β-Catenin Signaling. *Int. J. Mol. Sci.* 2020, *21*, 6357

**DOI:** 10.3390/ijms21228850

**Published:** 2020-11-23

**Authors:** Hui Shi, Luping Zhao, Xinlin Guo, Runping Fang, Hui Zhang, Guanjun Dong, Jia Fu, Fenglian Yan, Junfeng Zhang, Zhaochen Ning, Qun Ma, Zhihua Li, Chunxia Li, Jun Dai, Chuanping Si, Huabao Xiong

**Affiliations:** 1 Institute of Immunology and Molecular Medicine, Jining Medical University, Jining 272067, China; 8858shihui@mail.jnmc.edu.cn (H.S.); zhanghui1024@mail.jnmc.edu.cn (H.Z.); guanjun0323@mail.jnmc.edu.cn (G.D.); fujia730511@163.com (J.F.); yflian1117@mail.jnmc.edu.cn (F.Y.); zjfart001@163.com (J.Z.); ningzc@mail.jnmc.edu.cn (Z.N.); maqun@mail.jnmc.edu.cn (Q.M.); coco6016@mail.jnmc.edu.cn (Z.L.); xiachun1113@mail.jnmc.edu.cn (C.L.); immunedai@mail.jnmc.edu.cn (J.D.); 2 Institute of Basic Medical College, Jining Medical University, Jining 272067, China; zpersistence@163.com (L.Z.); gxlupupup@163.com (X.G.); 3 State Key Laboratory of Medicinal Chemical Biology, Department of Biochemistry, College of Life Sciences, Nankai University, Tianjin 300071, China; rpfang@163.com

The authors wish to make the following correction to this paper [1]. The reason for the correction is an error in representing β-catenin immunohistochemical staining pictures in the old version of Figure 6F. The mistake was due to mislinking two pictures from the same slide for two different groups. They should be replaced with the correct new figure (Figure 1).

The correction does not change the conclusions of this manuscript. The authors would like to apologize for any inconvenience caused to the readers by these changes.

## Figures and Tables

**Figure 1:**
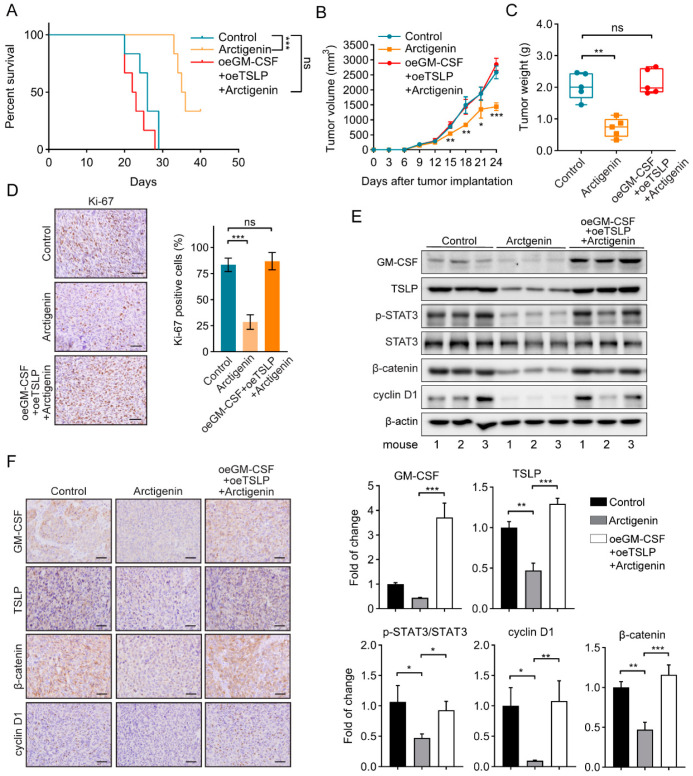
Arctigenin inhibits breast cancer progression via decreasing GM-CSF and TSLP in vivo. (**A**) Kaplan–Meier survival curves for mice injected with the indicated 4T1 cells and/or treated with arctigenin. oe, overexpression. n = 6 mice/group. *** *p* < 0.01, ns, not significant, *p* > 0.05 (Log Rank test). (**B**) The tumor volume of 4T1 tumors separated from mice of each group. (**C**) The tumor weight of separated 4T1 tumors in each group. (**D**) Representative Ki-67 staining (left panel) and statistics (right panel) of 4T1 tumors from the indicated mice. Scale bar, 100 μm. (**E**) The levels of GM-CSF, TSLP, p-STAT3, STAT3, β-catenin and cyclin D1 of the indicated tumors from representative mice were analyzed by Western blotting analysis. Mouse number was indicated at bottom. (**F**) Representative immunohistological staining of GM-CSF, TSLP, β-catenin and cyclin D1 in the indicated tumor sections. Scale bar, 100 μm. For (**B**)–(**D**) (right panel) and (**E**), the data are shown as mean ± SD. Statistically significant differences are indicated: * *p* < 0.05, ** *p* < 0.01, *** *p* < 0.001, ns, not significant, and *p* > 0.05 (One-way ANOVA).
