# Supplementary material for: Arctigenin Attenuates Breast Cancer Progression through Decreasing GM-CSF/TSLP/STAT3/β-Catenin Signaling
Source: Int J Mol Sci. 2020 Sep 2;21(17):6357. doi: 10.3390/ijms21176357 (PMC7503539; doi:10.3390/ijms21176357)
Supplement: Supplementary file 1 [file ijms-21-06357-s001.pdf]

Supporting Information for

Arctigenin attenuates breast cancer progression through decreasing GM-CSF/TSLP/STAT3/β-catenin signaling

Hui Shi [1], Luping zhao [2], Xinlin Guo [2], Runping Fang [3], Hui Zhang [1], Guanjun Dong [1], Jia Fu [1], Fenglian Yan [1], Junfeng Zhang [1], Zhaochen Ning [1], Qun Ma [1], Zhihua Li [1], Chunxia Li [1], Jun Dai [1], Chuanping Si [1,*] and Huabao Xiong [1,*]

[1] Institute of Immunology and Molecular Medicine, Jining Medical University, Shandong, 272067, China; xionghuabao@mail.jnmc.edu.cn (H.X.); chpsi@mail.jnmc.edu.cn (C.S.); 8858shihui@mail.jnmc.edu.cn (H.S.); zhanghui1024@mail.jnmc.edu.cn (H.Z.); guanjun0323@mail.jnmc.edu.cn (G.D.); fujia730511@163.com (J.F.); yflian1117@mail.jnmc.edu.cn (F.Y.); zjfart001@163.com (J.Z.); ningzc@mail.jnmc.edu.cn (Z.N.); maqun@mail.jnmc.edu.cn (Q.M.); coco6016@mail.jnmc.edu.cn (Z.L.); xiachun1113@mail.jnmc.edu.com (C.L.); immunedai@mail.jnmc.edu.cn (J.D.)

[2] Institute of Basic Medical College, Jining Medical University, Shandong, 272067, China; zpersistence@163.com (L.Z.); gxlupupup@163.com (X.G.)

[3] State Key Laboratory of Medicinal Chemical Biology, Department of Biochemistry, College of Life Sciences, Nankai University, Tianjin 300071, P.R. China; rpfang@163.com (R.F.)

* Correspondence: xionghuabao@mail.jnmc.edu.cn; Tel.: (+86(0537)3616283) (H.X.); chpsi@mail.jnmc.edu.cn
Tel.: (+86(0537)3616286)

This file contains:

Figure S1. Arctigenin suppresses GM-CSF and TSLP expression in breast cancer cells

Figure S2. Arctigenin inhibits breast cancer cells proliferation, invasion and stemness through decreasing GM-CSF and TSLP

Figure S3. Arctigenin inhibits STAT3/β-catenin signaling through decreasing GM-CSF and TSLP

Table S1. List of antibodies used in this paper.

Table S2. The characterization of the Mouse Cytokine Array C1000.

Table S3. List of primers used in this paper.

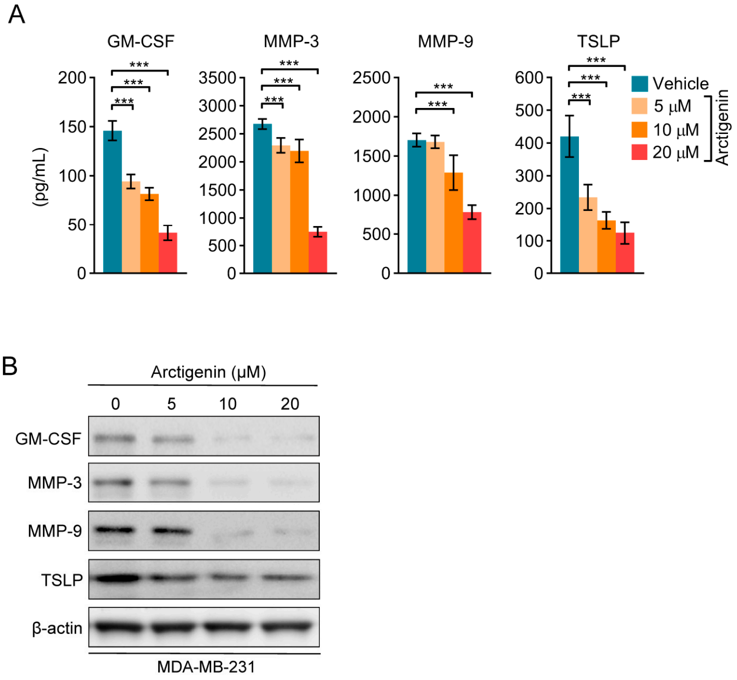

Figure S1. Arctigenin suppresses GM-CSF and TSLP expression in breast cancer cells. (**A**) Quantification of GM-CSF, MMP-3, MMP-9 and TSLP in conditioned medium from MDA-MB-231 cells in the presence or absence of indicated concentrations of arctigenin. Values represent mean ± SEM of three independent experiments. Statistically significant differences are indicated: ***P<0.001 (One-way ANOVA). (**B**) Western blot analysis of GM-CSF, MMP-3, MMP-9 and TSLP in MDA-MB-231 cells treated with arctigenin at indicated concentration.

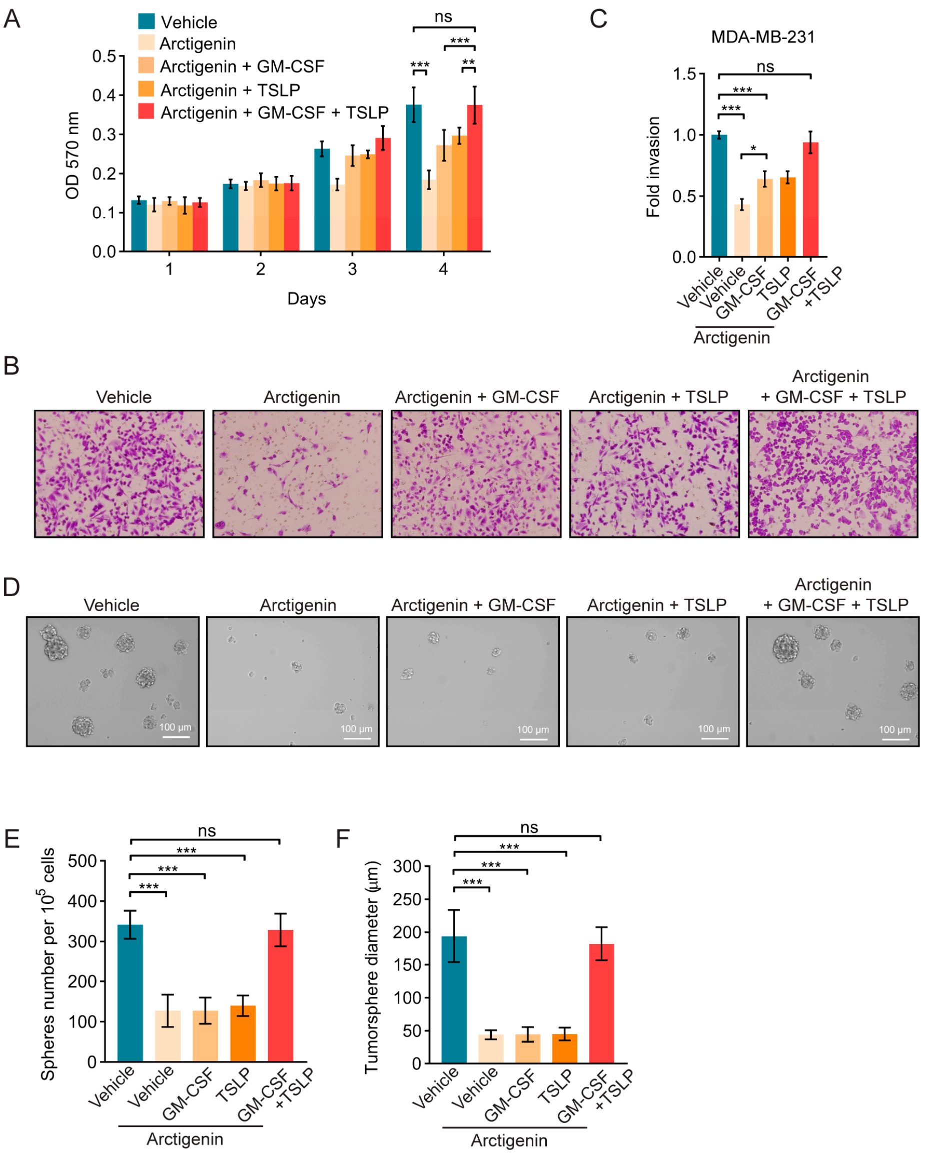

Figure S2. Arctigenin inhibits breast cancer cells proliferation, invasion and stemness through decreasing GM-CSF and TSLP. (**A**) Cell proliferation of MDA-MB-231 cells were treated with arctigenin (20 μM) in the presence or absence of GM-CSF (1ng/mL) and TSLP (100ng/mL). (**B**, **C**) Representative images (**B**) and statistics (**C**) of the invaded MDA-MB-231 cells treated with arctigenin (20 μM) in the presence or absence of GM-CSF and TSLP. (**D**) Representative images of the tumorsphere formation in MDA-MB-231 cells in the presence or absence of GM-CSF and TSLP. (**E**, **F**) Quantification of tumorspheres formation efficiency (**E**) and tumorsphere diameter (**F**). For **A**, **C**, **E** and **F**, the data are shown as means ± SD, ns, not significant, P>0.05, **P<0.01, ***P<0.001 (One-way ANOVA). Values represent mean ± SEM of three independent experiments.

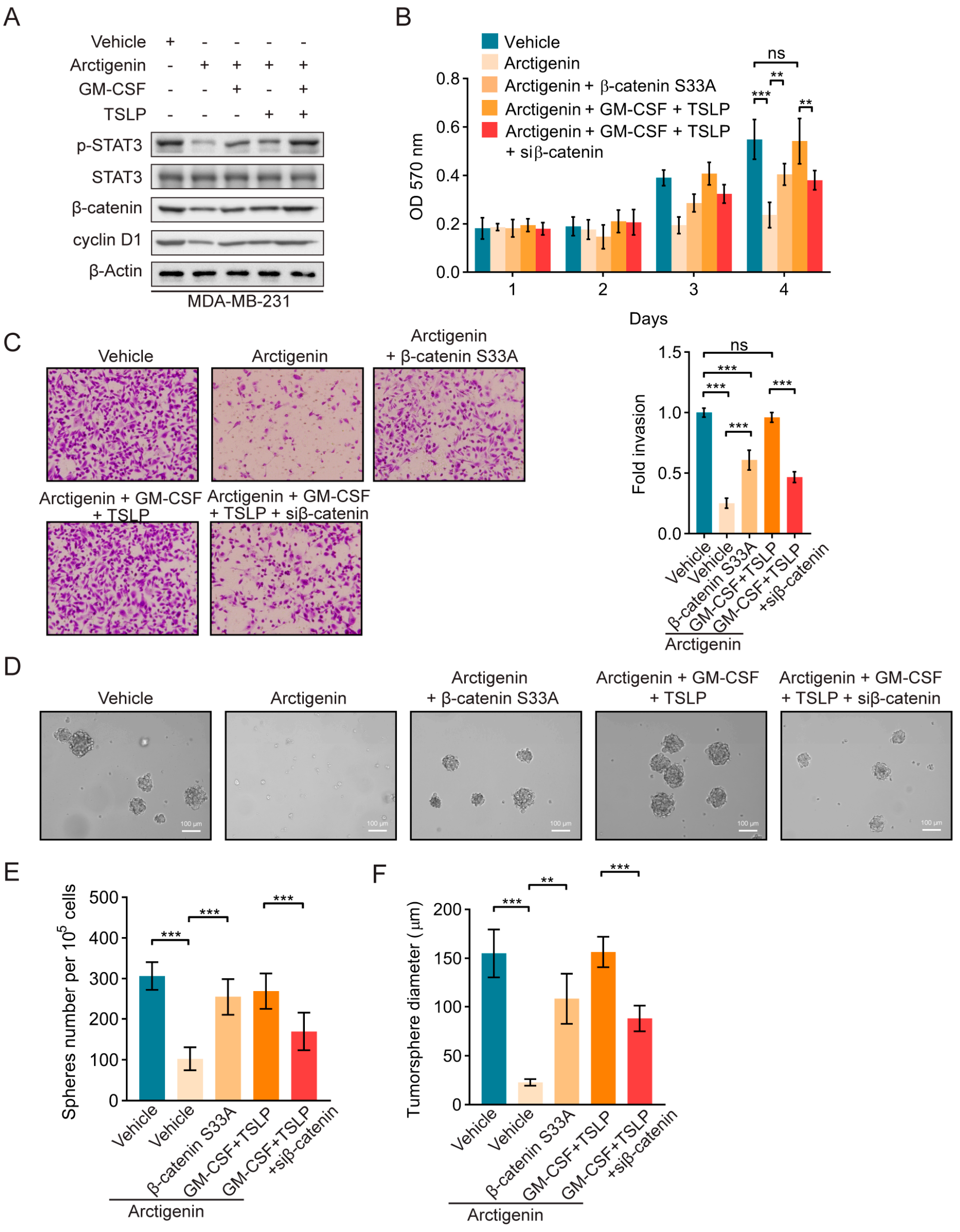

Figure S3. Arctigenin inhibits STAT3/β-catenin signaling through decreasing GM-CSF and TSLP. (**A**) The expression levels of phosphorylated STAT3 (p-STAT3), STAT3, β-catenin and cyclin D1 in MDA-MB-231 cells were analyzed by Western blotting with the indicated treatments. (**B**) Cell proliferation analysis of MDA-MB-231 cells treated with arctigenin (20 μM) in the presence or absence of GM-CSF (1 ng/mL), TSLP (100 ng/mL), or transfectd with β-catenin S33A construct or siRNA of β-catenin. (**C**) Representative images (left) and statistics (right) of the invaded MDA-MB-231 cells with the indicated treatments. (**D**) Representative images of the tumorsphere in MDA-MB-231 cells after indicated treatments. (**E**, **F**) Quantification of tumorspheres formation efficiency (E) and tumorsphere diameter (**F**). For B, **C**, **E**, and **F**, ns, not significant, P>0.05, **P<0.01, ***P<0.001 (One-way ANOVA). Values represent mean ± SEM of three independent experiments.

Table S1. List of antibodies used in this paper.

Antibodies	Source	Identifier
Antibodies for Western blot		
anti-GM-CSF	Abcam	Cat#ab9741
anti-MMP-3	Proteintech	Cat#17873-1-AP
anti-MMP-9	Proteintech	Cat#10375-2-AP
anti-TSLP	Abcam	Cat#ab188766
anti-p65	Cell Signaling Technology	Cat#8242
anti-STAT3	Cell Signaling Technology	Cat#4904
anti-phospho-STAT3(Y705)	Cell Signaling Technology	Cat#9145
anti-β-catenin	Cell Signaling Technology	Cat#8480
anti-Cyclin D1	Cell Signaling Technology	Cat#2978
anti-Histone H3	Cell Signaling Technology	Cat#4499
anti-β-actin	Sigma-Aldrich	Cat#SAB2100037
Antibodies for Immunofluorescence & Immunohistochemical staining		
anti-p65	Cell Signaling Technology	Cat#8242
anti-β-catenin	Cell Signaling Technology	Cat#8480
anti-Cyclin D1	Cell Signaling Technology	Cat#2978
anti-GM-CSF	Abcam	Cat#ab9741
anti-TSLP	Abcam	Cat#ab188766
anti-Ki-67	Abcam	Cat#ab16667

Table S2. The characterization of the Mouse Cytokine Array C1000 (C3 and C4).

AAM-CYT-3(C3)

	A	B	C	D	E	F	G	H	I	J	K	L	M	N
1	POS	POS	NEG	NEG	Blank	Axl	BLC	CD30L	CD30T	CD40	CRG-2	CTACK	CXCL16	Eotaxin
2	POS	POS	NEG	NEG	Blank	Axl	BLC	CD30L	CD30T	CD40	CRG-2	CTACK	CXCL16	Eotaxin
3	Eotoxin-2	Fas-ligand	Fract-alKine	GCSF	GM-CSF	IFN gamma	IGFBP-3	IGFBP-5	IGFBP-6	IL-1 alpha	IL-1 beta	IL-2	IL-3	IL-3 R beta
4	Eotoxin-2	Fas-ligand	Fract-alKine	GCSF	GM-CSF	IFN gamma	IGFBP-3	IGFBP-5	IGFBP-6	IL-1 alpha	IL-1 beta	IL-2	IL-3	IL-3 R beta
5	IL-4	IL-5	IL-6	IL-9	IL-10	IL-12 p40/p70	IL-12 p70	IL-13	IL-17A	KC	Leptin R	Leptin	LIX	L-Selectin
6	IL-4	IL-5	IL-6	IL-9	IL-10	IL-12 p40/p70	IL-12 p70	IL-13	IL-17A	KC	Leptin R	Leptin	LIX	L-Selectin
7	Lymphotactin	MCP1	MCP-5	M-CSF	MIG	MIP-1 alpha	MIP-1 gamma	MIP-2	MIP-3 beta	MIP-3 alpha	PF-4	P-Selectin	RANTES	SCF
8	Lymphotactin	MCP1	MCP-5	M-CSF	MIG	MIP-1 alpha	MIP-1 gamma	MIP-2	MIP-3 beta	MIP-3 alpha	PF-4	P-Selectin	RANTES	SCF
9	SDF-1 alpha	TARC	TCA-3	TECK	TIMP-1	TNF-alpha	sTNF RI	sTNF RII	TPO	VCAM-1	VEGF	Blank	Blank	POS
10	SDF-1 alpha	TARC	TCA-3	TECK	TIMP-1	TNF-alpha	sTNF RI	sTNF RII	TPO	VCAM-1	VEGF	Blank	Blank	POS

AAM-CYT-4(C4)

	A	B	C	D	E	F	G	H	I	J	K	L
1	POS	POS	NEG	NEG	Blank	bFGF	CD26	Dtk	E-Selectin	Fc-gamma	Flt-3L	GITR
2	POS	POS	NEG	NEG	Blank	bFGF	CD26	Dtk	E-Selectin	Fc-gamma	Flt-3L	GITR
3	HGFR	ICAM	IGFBP-2	IGF-1	IGF-2	IL-15	IL-17RB	IL-7	I-TAC	Lungkine	MDC	MMP-2
4	HGFR	ICAM	IGFBP-2	IGF-1	IGF-2	IL-15	IL-17RB	IL-7	I-TAC	Lungkine	MDC	MMP-2
5	MMP-3	OPN	OPG	Pro-MMp-9	Resistin	Shh-N	TCK-1	TIMP-2	TRANCE	TROY	TSLP	VEGFR1
6	MMP-3	OPN	OPG	Pro-MMp-9	Resistin	Shh-N	TCK-1	TIMP-2	TRANCE	TROY	TSLP	VEGFR1
7	VEGFR2	VEGFR3	VEGF-D	BLANK	BLANK	BLANK	BLANK	BLANK	BLANK	BLANK	BLANK	POS
8	VEGFR2	VEGFR3	VEGF-D	BLANK	BLANK	BLANK	BLANK	BLANK	BLANK	BLANK	BLANK	POS

Table S3. List of primers used in this paper.

Gene	Primer	Sequence (5′-3′)
**Primers for GM-CSF promoter**		
pGL3-Csf2	forward	CACTCAGTATCTCCCAAACC
	reverse	CAGGACCTTAGCCTTTCTC
pGL3-Csf2-MUT		
Site 1	forward	CATGTATAGCTGATAAGGGCCATTCGATTCCACAACTCAGGTAGTTC
	reverse	GAACTACCTGAGTTGTGGAATCGAATGGCCCTTATCAGCTATACATG
Site 2	forward	GGGCCAGGAGATTCCACAACTCATTCAGTTCCCCCGCC
	reverse	GGCGGGGGAACTGAATGAGTTGTGGAATCTCCTGGCCC
pGL3-Tslp	forward	ACTGCGGGTGTTAGAAGG
	reverse	AAGACTGTGCTCGGGTAT
pGL3-Tslp-MUT		
	forward	AAAATAAAATAAAATAAAAGGAAAGGAAAAAGGTGAGTTCAATTCCTGATGATTTTGCTAAAGTTAAAATTCCATAG
	reverse	CTATGGAATTTTAACTTTAGCAAAATCATCAGGAATTGAACTCACCTTTTTCCTTTCCTTTTATTTTATTTTATTTT
**Primers for qRT-PCR**		
Csf2	forward	GCGGGTCTGCACACATGTTA
	reverse	GCCATCAAAGAAGCCCTGAA
Mmp-3	forward	AGGGATGATGAGCTGGTATG
	reverse	AACACCACACCTGGGCTTAT
pro-Mmp-9	forward	GCCGACTTTTGTGGTCTTCC
	reverse	GGTACAAGTATGCCTCTGCCA
Tslp	forward	GGACCACTGGTGTTTATTCT
	reverse	CAGGGTTTAGATGCTGTCAT
Axin2	forward	AACCTATGCCCGTTTCCTCTA
	reverse	GAGTGTAAAGACTTGGTCCACC
Myc	forward	GCTGGACACGCTGACGAAA
	reverse	TCTAGGCGAAGCAGCTCTATTT
Lef1	forward	GCCACCGATGAGATGATCCC
	reverse	TTGATGTCGGCTAAGTCGCC
Ccnd1	forward	GGCAGCCCCAACAACTTC
	reverse	TCCCGCCTGCCCGGTGG
Nanog	forward	TGAGTGTGGGTCTTCCTGGT
	reverse	CAGGCATTGATGAGGCGTTC
Oct4	forward	GAAGCAGAAGAGGATCACCT

	reverse	TTCTTAAGGCTGAGCTGCAAG
Actb	forward	TCATGAAGTGTGACGTTGACA
	reverse	CCTAGAAGCATTTGCGGTG
